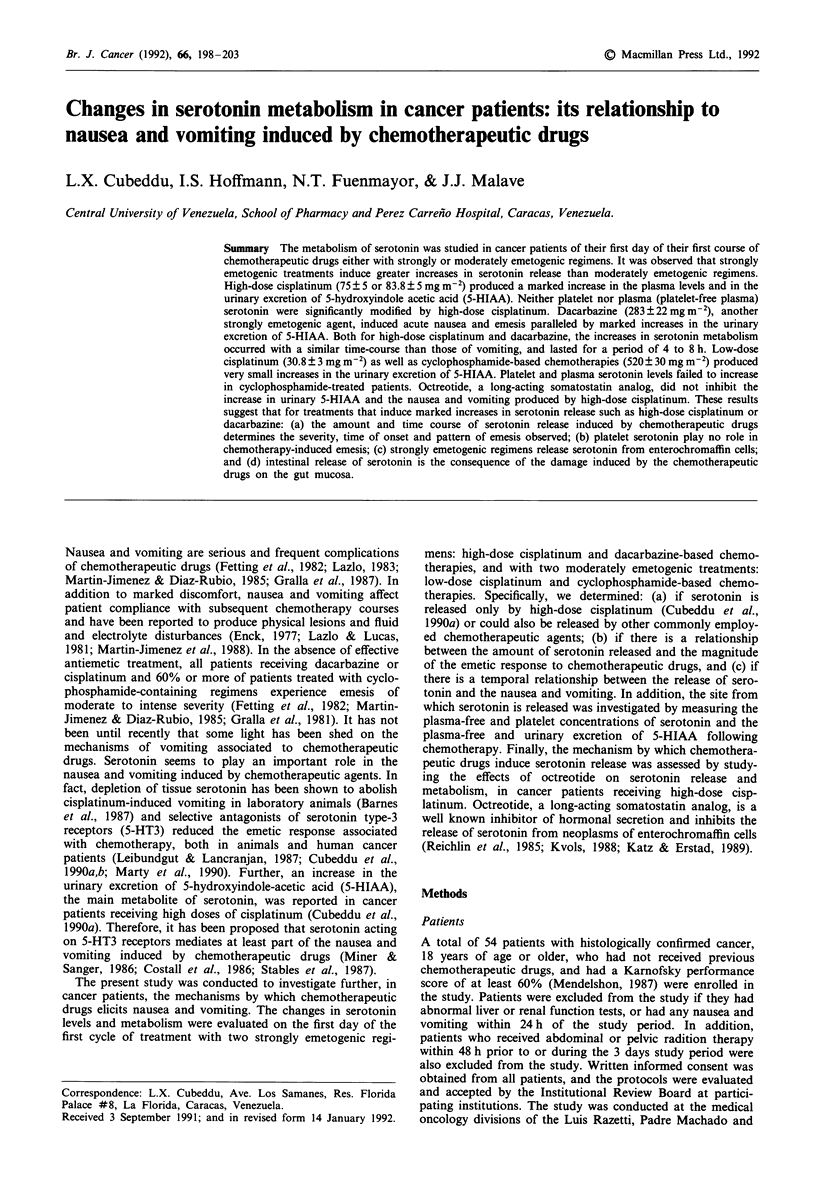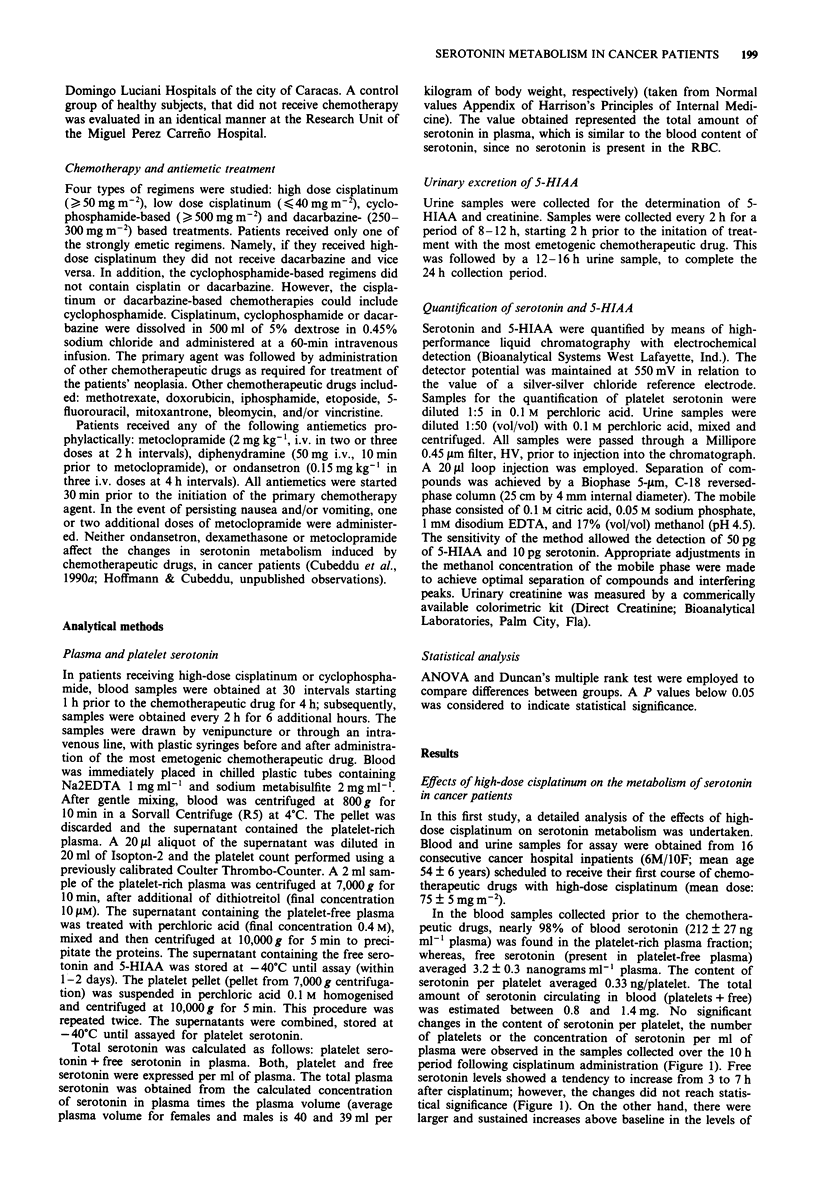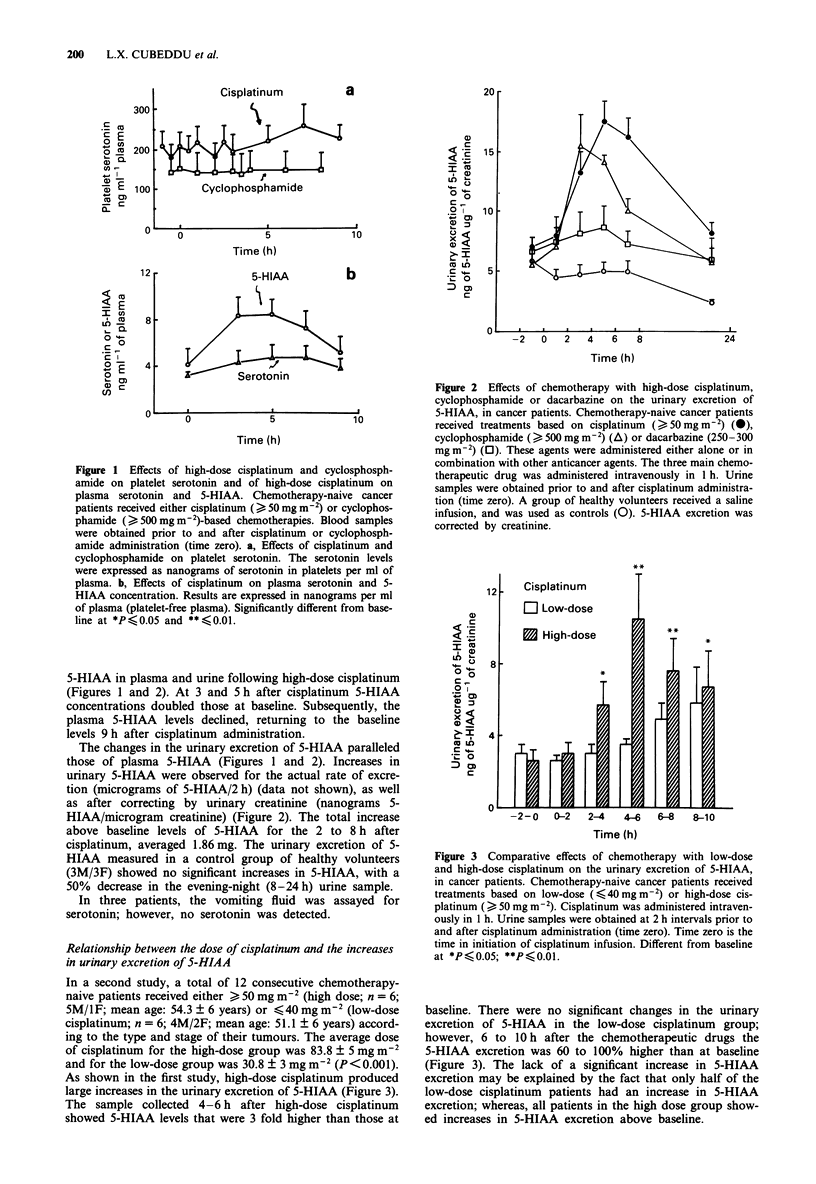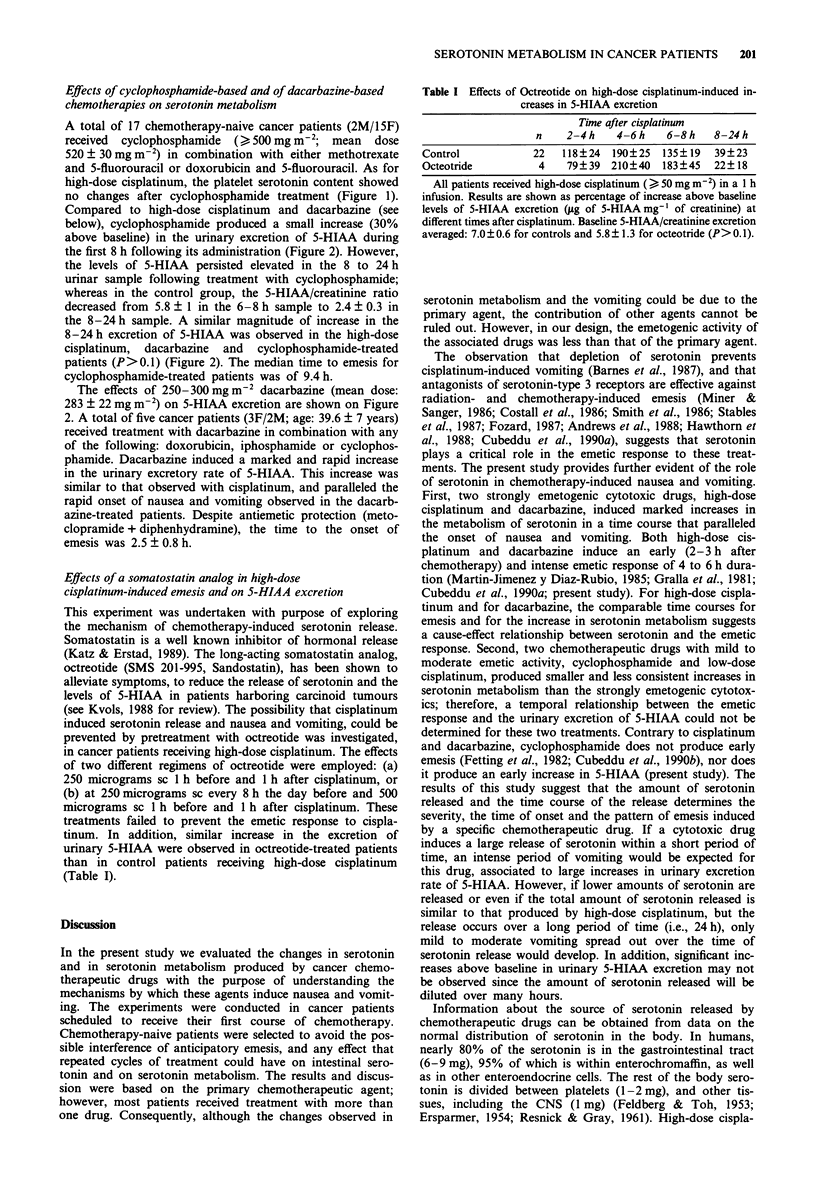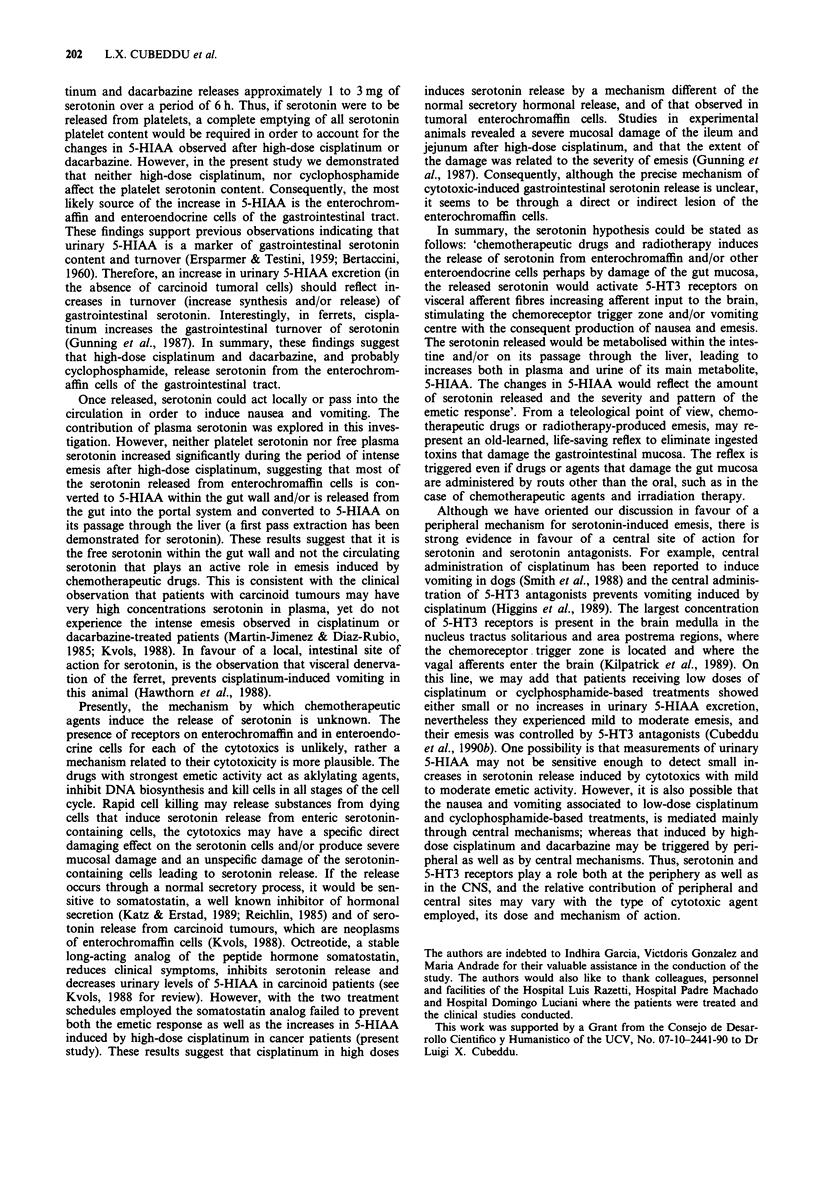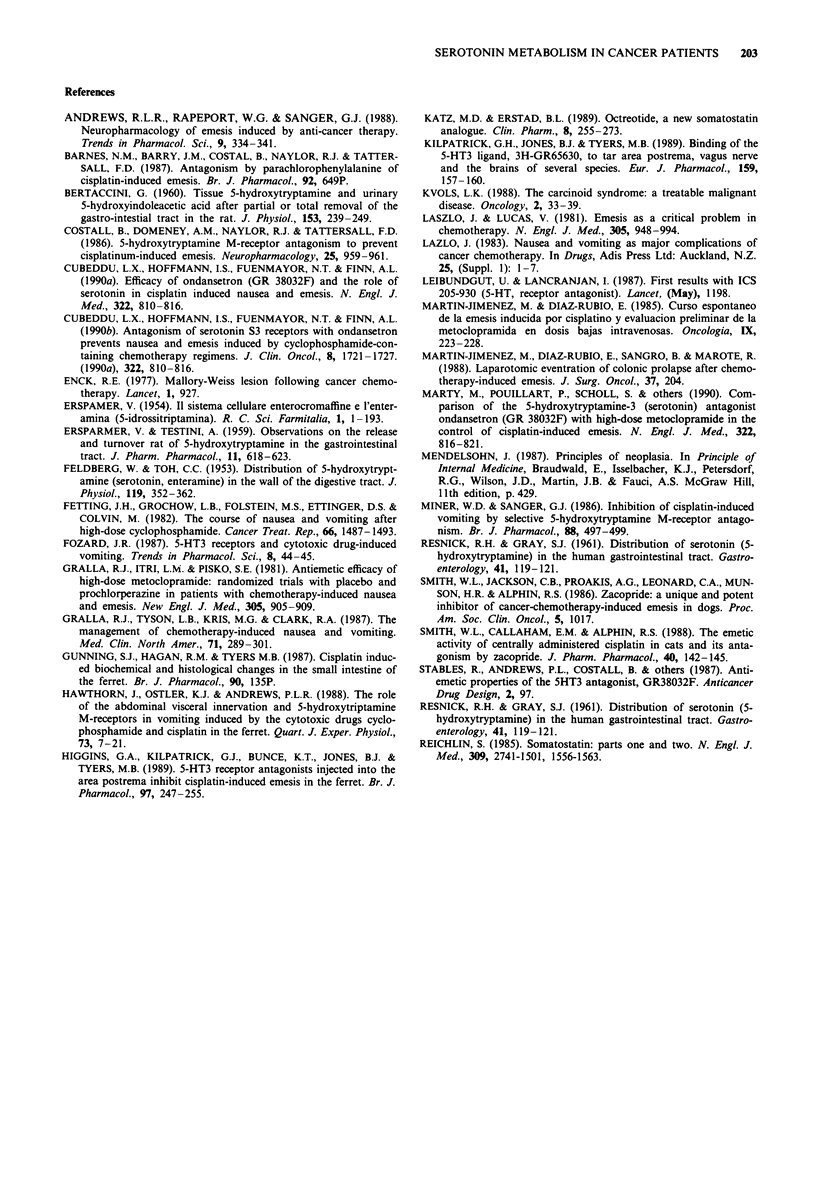# Changes in serotonin metabolism in cancer patients: its relationship to nausea and vomiting induced by chemotherapeutic drugs.

**DOI:** 10.1038/bjc.1992.242

**Published:** 1992-07

**Authors:** L. X. Cubeddu, I. S. Hoffmann, N. T. Fuenmayor, J. J. Malave

**Affiliations:** Central University of Venezuela, School of Pharmacy, Caracas.

## Abstract

The metabolism of serotonin was studied in cancer patients of their first day of their first course of chemotherapeutic drugs either with strongly or moderately emetogenic regimens. It was observed that strongly emetogenic treatments induce greater increases in serotonin release than moderately emetogenic regimens. High-dose cisplatinum (75 +/- 5 or 83.8 +/- 5 mg m-2) produced a marked increase in the plasma levels and in the urinary excretion of 5-hydroxyindole acetic acid (5-HIAA). Neither platelet nor plasma (platelet-free plasma) serotonin were significantly modified by high-dose cisplatinum. Dacarbazine (283 +/- 22 mg m-2), another strongly emetogenic agent, induced acute nausea and emesis paralleled by marked increases in the urinary excretion of 5-HIAA. Both for high-dose cisplatinum and dacarbazine, the increases in serotonin metabolism occurred with a similar time-course than those of vomiting, and lasted for a period of 4 to 8 h. Low-dose cisplatinum (30.8 +/- 3 mg m-2) as well as cyclophosphamide-based chemotherapies (520 +/- 30 mg m-2) produced very small increases in the urinary excretion of 5-HIAA. Platelet and plasma serotonin levels failed to increase in cyclophosphamide-treated patients. Octreotide, a long-acting somatostatin analog, did not inhibit the increase in urinary 5-HIAA and the nausea and vomiting produced by high-dose cisplatinum. These results suggest that for treatments that induce marked increases in serotonin release such as high-dose cisplatinum or dacarbazine: (a) the amount and time course of serotonin release induced by chemotherapeutic drugs determines the severity, time of onset and pattern of emesis observed; (b) platelet serotonin play no role in chemotherapy-induced emesis; (c) strongly emetogenic regimens release serotonin from enterochromaffin cells; and (d) intestinal release of serotonin is the consequence of the damage induced by the chemotherapeutic drugs on the gut mucosa.


*Br. J. Cancer* (1992), **66**, 198–203 © Macmillan Press Ltd., 1992

### Changes in serotonin metabolism in cancer patients: its relationship to nausea and vomiting induced by chemotherapeutic drugs

L.X. Cubeddu, I.S. Hoffmann, N.T. Fuenmayor, & J.J. Malave

*Central University of Venezuela, School of Pharmacy and Perez Carreño Hospital, Caracas, Venezuela.*

**Summary** The metabolism of serotonin was studied in cancer patients of their first day of their first course of chemotherapeutic drugs either with strongly or moderately emetogenic regimens. It was observed that strongly emetogenic treatments induce greater increases in serotonin release than moderately emetogenic regimens. High-dose cisplatinum ($75 \pm 5$ or $83.8 \pm 5$ mg m$^{-2}$) produced a marked increase in the plasma levels and in the urinary excretion of 5-hydroxyindole acetic acid (5-HIAA). Neither platelet nor plasma (platelet-free plasma) serotonin were significantly modified by high-dose cisplatinum. Dacarbazine ($283 \pm 22$ mg m$^{-2}$), another strongly emetogenic agent, induced acute nausea and emesis paralleled by marked increases in the urinary excretion of 5-HIAA. Both for high-dose cisplatinum and dacarbazine, the increases in serotonin metabolism occurred with a similar time-course than those of vomiting, and lasted for a period of 4 to 8 h. Low-dose cisplatinum ($30.8 \pm 3$ mg m$^{-2}$) as well as cyclophosphamide-based chemotherapies ($520 \pm 30$ mg m$^{-2}$) produced very small increases in the urinary excretion of 5-HIAA. Platelet and plasma serotonin levels failed to increase in cyclophosphamide-treated patients. Octreotide, a long-acting somatostatin analog, did not inhibit the increase in urinary 5-HIAA and the nausea and vomiting produced by high-dose cisplatinum. These results suggest that for treatments that induce marked increases in serotonin release such as high-dose cisplatinum or dacarbazine: (a) the amount and time course of serotonin release induced by chemotherapeutic drugs determines the severity, time of onset and pattern of emesis observed; (b) platelet serotonin play no role in chemotherapy-induced emesis; (c) strongly emetogenic regimens release serotonin from enterochromaffin cells; and (d) intestinal release of serotonin is the consequence of the damage induced by the chemotherapeutic drugs on the gut mucosa.

Nausea and vomiting are serious and frequent complications of chemotherapeutic drugs (Fetting *et al.*, 1982; Lazlo, 1983; Martin-Jimenez & Diaz-Rubio, 1985; Gralla *et al.*, 1987). In addition to marked discomfort, nausea and vomiting affect patient compliance with subsequent chemotherapy courses and have been reported to produce physical lesions and fluid and electrolyte disturbances (Enck, 1977; Lazlo & Lucas, 1981; Martin-Jimenez *et al.*, 1988). In the absence of effective antiemetic treatment, all patients receiving dacarbazine or cisplatinum and 60% or more of patients treated with cyclophosphamide-containing regimens experience emesis of moderate to intense severity (Fetting *et al.*, 1982; Martin-Jimenez & Diaz-Rubio, 1985; Gralla *et al.*, 1981). It has not been until recently that some light has been shed on the mechanisms of vomiting associated to chemotherapeutic drugs. Serotonin seems to play an important role in the nausea and vomiting induced by chemotherapeutic agents. In fact, depletion of tissue serotonin has been shown to abolish cisplatinum-induced vomiting in laboratory animals (Barnes *et al.*, 1987) and selective antagonists of serotonin type-3 receptors (5-HT3) reduced the emetic response associated with chemotherapy, both in animals and human cancer patients (Leibundgut & Lancranjan, 1987; Cubeddu *et al.*, 1990*a,b*; Marty *et al.*, 1990). Further, an increase in the urinary excretion of 5-hydroxyindole-acetic acid (5-HIAA), the main metabolite of serotonin, was reported in cancer patients receiving high doses of cisplatinum (Cubeddu *et al.*, 1990*a*). Therefore, it has been proposed that serotonin acting on 5-HT3 receptors mediates at least part of the nausea and vomiting induced by chemotherapeutic drugs (Miner & Sanger, 1986; Costall *et al.*, 1986; Stables *et al.*, 1987).

The present study was conducted to investigate further, in cancer patients, the mechanisms by which chemotherapeutic drugs elicits nausea and vomiting. The changes in serotonin levels and metabolism were evaluated on the first day of the first cycle of treatment with two strongly emetogenic regimens: high-dose cisplatinum and dacarbazine-based chemotherapies, and with two moderately emetogenic treatments: low-dose cisplatinum and cyclophosphamide-based chemotherapies. Specifically, we determined: (a) if serotonin is released only by high-dose cisplatinum (Cubeddu *et al.*, 1990*a*) or could also be released by other commonly employed chemotherapeutic agents; (b) if there is a relationship between the amount of serotonin released and the magnitude of the emetic response to chemotherapeutic drugs, and (c) if there is a temporal relationship between the release of serotonin and the nausea and vomiting. In addition, the site from which serotonin is released was investigated by measuring the plasma-free and platelet concentrations of serotonin and the plasma-free and urinary excretion of 5-HIAA following chemotherapy. Finally, the mechanism by which chemotherapeutic drugs induce serotonin release was assessed by studying the effects of octreotide on serotonin release and metabolism, in cancer patients receiving high-dose cisplatinum. Octreotide, a long-acting somatostatin analog, is a well known inhibitor of hormonal secretion and inhibits the release of serotonin from neoplasms of enterochromaffin cells (Reichlin *et al.*, 1985; Kvols, 1988; Katz & Erstad, 1989).

### Methods

### *Patients*

A total of 54 patients with histologically confirmed cancer, 18 years of age or older, who had not received previous chemotherapeutic drugs, and had a Karnofsky performance score of at least 60% (Mendelshon, 1987) were enrolled in the study. Patients were excluded from the study if they had abnormal liver or renal function tests, or had any nausea and vomiting within 24 h of the study period. In addition, patients who received abdominal or pelvic radition therapy within 48 h prior to or during the 3 days study period were also excluded from the study. Written informed consent was obtained from all patients, and the protocols were evaluated and accepted by the Institutional Review Board at participating institutions. The study was conducted at the medical oncology divisions of the Luis Razetti, Padre Machado and

Correspondence: L.X. Cubeddu, Ave. Los Samanes, Res. Florida Palace #8, La Florida, Caracas, Venezuela.
Received 3 September 1991; and in revised form 14 January 1992.

Domingo Luciani Hospitals of the city of Caracas. A control group of healthy subjects, that did not receive chemotherapy was evaluated in an identical manner at the Research Unit of the Miguel Perez Carreño Hospital.

*Chemotherapy and antiemetic treatment*

Four types of regimens were studied: high dose cisplatinum ($\geqslant 50$ mg m$^{-2}$), low dose cisplatinum ($\leqslant 40$ mg m$^{-2}$), cyclophosphamide-based ($\geqslant 500$ mg m$^{-2}$) and dacarbazine- (250–300 mg m$^{-2}$) based treatments. Patients received only one of the strongly emetic regimens. Namely, if they received high-dose cisplatinum they did not receive dacarbazine and vice versa. In addition, the cyclophosphamide-based regimens did not contain cisplatin or dacarbazine. However, the cisplatinum or dacarbazine-based chemotherapies could include cyclophosphamide. Cisplatinum, cyclophosphamide or dacarbazine were dissolved in 500 ml of 5% dextrose in 0.45% sodium chloride and administered at a 60-min intravenous infusion. The primary agent was followed by administration of other chemotherapeutic drugs as required for treatment of the patients' neoplasia. Other chemotherapeutic drugs included: methotrexate, doxorubicin, iphosphamide, etoposide, 5-fluorouracil, mitoxantrone, bleomycin, and/or vincristine.

Patients received any of the following antiemetics prophylactically: metoclopramide (2 mg kg$^{-1}$, i.v. in two or three doses at 2 h intervals), diphenydramine (50 mg i.v., 10 min prior to metoclopramide), or ondansetron (0.15 mg kg$^{-1}$ in three i.v. doses at 4 h intervals). All antiemetics were started 30 min prior to the initiation of the primary chemotherapy agent. In the event of persisting nausea and/or vomiting, one or two additional doses of metoclopramide were administered. Neither ondansetron, dexamethasone or metoclopramide affect the changes in serotonin metabolism induced by chemotherapeutic drugs, in cancer patients (Cubeddu *et al.*, 1990*a*; Hoffmann & Cubeddu, unpublished observations).

### Analytical methods

*Plasma and platelet serotonin*

In patients receiving high-dose cisplatinum or cyclophosphamide, blood samples were obtained at 30 intervals starting 1 h prior to the chemotherapeutic drug for 4 h; subsequently, samples were obtained every 2 h for 6 additional hours. The samples were drawn by venipuncture or through an intravenous line, with plastic syringes before and after administration of the most emetogenic chemotherapeutic drug. Blood was immediately placed in chilled plastic tubes containing Na2EDTA 1 mg ml$^{-1}$ and sodium metabisulfite 2 mg ml$^{-1}$. After gentle mixing, blood was centrifuged at 800 *g* for 10 min in a Sorvall Centrifuge (R5) at 4°C. The pellet was discarded and the supernatant contained the platelet-rich plasma. A 20 $\mu$l aliquot of the supernatant was diluted in 20 ml of Isopton-2 and the platelet count performed using a previously calibrated Coulter Thrombo-Counter. A 2 ml sample of the platelet-rich plasma was centrifuged at 7,000 *g* for 10 min, after additional of dithiotreitol (final concentration 10 $\mu$M). The supernatant containing the platelet-free plasma was treated with perchloric acid (final concentration 0.4 M), mixed and then centrifuged at 10,000 *g* for 5 min to precipitate the proteins. The supernatant containing the free serotonin and 5-HIAA was stored at −40°C until assay (within 1–2 days). The platelet pellet (pellet from 7,000 *g* centrifugation) was suspended in perchloric acid 0.1 M homogenised and centrifuged at 10,000 *g* for 5 min. This procedure was repeated twice. The supernatants were combined, stored at −40°C until assayed for platelet serotonin.

Total serotonin was calculated as follows: platelet serotonin + free serotonin in plasma. Both, platelet and free serotonin were expressed per ml of plasma. The total plasma serotonin was obtained from the calculated concentration of serotonin in plasma times the plasma volume (average plasma volume for females and males is 40 and 39 ml per kilogram of body weight, respectively) (taken from Normal values Appendix of Harrison's Principles of Internal Medicine). The value obtained represented the total amount of serotonin in plasma, which is similar to the blood content of serotonin, since no serotonin is present in the RBC.

*Urinary excretion of 5-HIAA*

Urine samples were collected for the determination of 5-HIAA and creatinine. Samples were collected every 2 h for a period of 8–12 h, starting 2 h prior to the initation of treatment with the most emetogenic chemotherapeutic drug. This was followed by a 12–16 h urine sample, to complete the 24 h collection period.

*Quantification of serotonin and 5-HIAA*

Serotonin and 5-HIAA were quantified by means of high-performance liquid chromatography with electrochemical detection (Bioanalytical Systems West Lafayette, Ind.). The detector potential was maintained at 550 mV in relation to the value of a silver-silver chloride reference electrode. Samples for the quantification of platelet serotonin were diluted 1:5 in 0.1 M perchloric acid. Urine samples were diluted 1:50 (vol/vol) with 0.1 M perchloric acid, mixed and centrifuged. All samples were passed through a Millipore 0.45 $\mu$m filter, HV, prior to injection into the chromatograph. A 20 $\mu$l loop injection was employed. Separation of compounds was achieved by a Biophase 5-$\mu$m, C-18 reversed-phase column (25 cm by 4 mm internal diameter). The mobile phase consisted of 0.1 M citric acid, 0.05 M sodium phosphate, 1 mM disodium EDTA, and 17% (vol/vol) methanol (pH 4.5). The sensitivity of the method allowed the detection of 50 pg of 5-HIAA and 10 pg serotonin. Appropriate adjustments in the methanol concentration of the mobile phase were made to achieve optimal separation of compounds and interfering peaks. Urinary creatinine was measured by a commerically available colorimetric kit (Direct Creatinine; Bioanalytical Laboratories, Palm City, Fla).

*Statistical analysis*

ANOVA and Duncan's multiple rank test were employed to compare differences between groups. A *P* values below 0.05 was considered to indicate statistical significance.

### Results

*Effects of high-dose cisplatinum on the metabolism of serotonin in cancer patients*

In this first study, a detailed analysis of the effects of high-dose cisplatinum on serotonin metabolism was undertaken. Blood and urine samples for assay were obtained from 16 consecutive cancer hospital inpatients (6M/10F; mean age $54 \pm 6$ years) scheduled to receive their first course of chemotherapeutic drugs with high-dose cisplatinum (mean dose: $75 \pm 5$ mg m$^{-2}$).

In the blood samples collected prior to the chemotherapeutic drugs, nearly 98% of blood serotonin ($212 \pm 27$ ng ml$^{-1}$ plasma) was found in the platelet-rich plasma fraction; whereas, free serotonin (present in platelet-free plasma) averaged $3.2 \pm 0.3$ nanograms ml$^{-1}$ plasma. The content of serotonin per platelet averaged 0.33 ng/platelet. The total amount of serotonin circulating in blood (platelets + free) was estimated between 0.8 and 1.4 mg. No significant changes in the content of serotonin per platelet, the number of platelets or the concentration of serotonin per ml of plasma were observed in the samples collected over the 10 h period following cisplatinum administration (Figure 1). Free serotonin levels showed a tendency to increase from 3 to 7 h after cisplatinum; however, the changes did not reach statistical significance (Figure 1). On the other hand, there were larger and sustained increases above baseline in the levels of

**Figure 1** Effects of high-dose cisplatinum and cyclosphosphamide on platelet serotonin and of high-dose cisplatinum on plasma serotonin and 5-HIAA. Chemotherapy-naive cancer patients received either cisplatinum ($\geqslant 50\ mg\ m^{-2}$) or cyclophosphamide ($\geqslant 500\ mg\ m^{-2}$)-based chemotherapies. Blood samples were obtained prior to and after cisplatinum or cyclophosphamide administration (time zero). **a**, Effects of cisplatinum and cyclophosphamide on platelet serotonin. The serotonin levels were expressed as nanograms of serotonin in platelets per ml of plasma. **b**, Effects of cisplatinum on plasma serotonin and 5-HIAA concentration. Results are expressed in nanograms per ml of plasma (platelet-free plasma). Significantly different from baseline at $^{*}P \leqslant 0.05$ and $^{**} \leqslant 0.01$.

5-HIAA in plasma and urine following high-dose cisplatinum (Figures 1 and 2). At 3 and 5 h after cisplatinum 5-HIAA concentrations doubled those at baseline. Subsequently, the plasma 5-HIAA levels declined, returning to the baseline levels 9 h after cisplatinum administration.

The changes in the urinary excretion of 5-HIAA paralleled those of plasma 5-HIAA (Figures 1 and 2). Increases in urinary 5-HIAA were observed for the actual rate of excretion (micrograms of 5-HIAA/2 h) (data not shown), as well as after correcting by urinary creatinine (nanograms 5-HIAA/microgram creatinine) (Figure 2). The total increase above baseline levels of 5-HIAA for the 2 to 8 h after cisplatinum, averaged 1.86 mg. The urinary excretion of 5-HIAA measured in a control group of healthy volunteers (3M/3F) showed no significant increases in 5-HIAA, with a 50% decrease in the evening-night (8–24 h) urine sample.

In three patients, the vomiting fluid was assayed for serotonin; however, no serotonin was detected.

### *Relationship between the dose of cisplatinum and the increases in urinary excretion of 5-HIAA*

In a second study, a total of 12 consecutive chemotherapy-naive patients received either $\geqslant 50\ mg\ m^{-2}$ (high dose; $n = 6$; 5M/1F; mean age: 54.3 ± 6 years) or $\leqslant 40\ mg\ m^{-2}$ (low-dose cisplatinum; $n = 6$; 4M/2F; mean age: 51.1 ± 6 years) according to the type and stage of their tumours. The average dose of cisplatinum for the high-dose group was $83.8 \pm 5\ mg\ m^{-2}$ and for the low-dose group was $30.8 \pm 3\ mg\ m^{-2}$ ($P < 0.001$). As shown in the first study, high-dose cisplatinum produced large increases in the urinary excretion of 5-HIAA (Figure 3). The sample collected 4–6 h after high-dose cisplatinum showed 5-HIAA levels that were 3 fold higher than those at

**Figure 2** Effects of chemotherapy with high-dose cisplatinum, cyclophosphamide or dacarbazine on the urinary excretion of 5-HIAA, in cancer patients. Chemotherapy-naive cancer patients received treatments based on cisplatinum ($\geqslant 50\ mg\ m^{-2}$) (●), cyclophosphamide ($\geqslant 500\ mg\ m^{-2}$) (△) or dacarbazine (250–300 $mg\ m^{-2}$) (□). These agents were administered either alone or in combination with other anticancer agents. The three main chemotherapeutic drug was administered intravenously in 1 h. Urine samples were obtained prior to and after cisplatinum administration (time zero). A group of healthy volunteers received a saline infusion, and was used as controls (○). 5-HIAA excretion was corrected by creatinine.

**Figure 3** Comparative effects of chemotherapy with low-dose and high-dose cisplatinum on the urinary excretion of 5-HIAA, in cancer patients. Chemotherapy-naive cancer patients received treatments based on low-dose ($\leqslant 40\ mg\ m^{-2}$) or high-dose cisplatinum ($\geqslant 50\ mg\ m^{-2}$). Cisplatinum was administered intravenously in 1 h. Urine samples were obtained at 2 h intervals prior to and after cisplatinum administration (time zero). Time zero is the time in initiation of cisplatinum infusion. Different from baseline at $^{*}P \leqslant 0.05$; $^{**}P \leqslant 0.01$.

baseline. There were no significant changes in the urinary excretion of 5-HIAA in the low-dose cisplatinum group; however, 6 to 10 h after the chemotherapeutic drugs the 5-HIAA excretion was 60 to 100% higher than at baseline (Figure 3). The lack of a significant increase in 5-HIAA excretion may be explained by the fact that only half of the low-dose cisplatinum patients had an increase in 5-HIAA excretion; whereas, all patients in the high dose group showed increases in 5-HIAA excretion above baseline.

*Effects of cyclophosphamide-based and of dacarbazine-based chemotherapies on serotonin metabolism*

A total of 17 chemotherapy-naive cancer patients (2M/15F) received cyclophosphamide ($\geqslant 500 \text{ mg m}^{-2}$; mean dose $520 \pm 30 \text{ mg m}^{-2}$) in combination with either methotrexate and 5-fluorouracil or doxorubicin and 5-fluorouracil. As for high-dose cisplatinum, the platelet serotonin content showed no changes after cyclophosphamide treatment (Figure 1). Compared to high-dose cisplatinum and dacarbazine (see below), cyclophosphamide produced a small increase (30% above baseline) in the urinary excretion of 5-HIAA during the first 8 h following its administration (Figure 2). However, the levels of 5-HIAA persisted elevated in the 8 to 24 h urinar sample following treatment with cyclophosphamide; whereas in the control group, the 5-HIAA/creatinine ratio decreased from $5.8 \pm 1$ in the 6–8 h sample to $2.4 \pm 0.3$ in the 8–24 h sample. A similar magnitude of increase in the 8–24 h excretion of 5-HIAA was observed in the high-dose cisplatinum, dacarbazine and cyclophosphamide-treated patients ($P > 0.1$) (Figure 2). The median time to emesis for cyclophosphamide-treated patients was of 9.4 h.

The effects of 250–300 $\text{mg m}^{-2}$ dacarbazine (mean dose: $283 \pm 22 \text{ mg m}^{-2}$) on 5-HIAA excretion are shown on Figure 2. A total of five cancer patients (3F/2M; age: $39.6 \pm 7$ years) received treatment with dacarbazine in combination with any of the following: doxorubicin, iphosphamide or cyclophosphamide. Dacarbazine induced a marked and rapid increase in the urinary excretory rate of 5-HIAA. This increase was similar to that observed with cisplatinum, and paralleled the rapid onset of nausea and vomiting observed in the dacarbazine-treated patients. Despite antiemetic protection (metoclopramide + diphenhydramine), the time to the onset of emesis was $2.5 \pm 0.8$ h.

*Effects of a somatostatin analog in high-dose cisplatinum-induced emesis and on 5-HIAA excretion*

This experiment was undertaken with purpose of exploring the mechanism of chemotherapy-induced serotonin release. Somatostatin is a well known inhibitor of hormonal release (Katz & Erstad, 1989). The long-acting somatostatin analog, octreotide (SMS 201-995, Sandostatin), has been shown to alleviate symptoms, to reduce the release of serotonin and the levels of 5-HIAA in patients harboring carcinoid tumours (see Kvols, 1988 for review). The possibility that cisplatinum induced serotonin release and nausea and vomiting, could be prevented by pretreatment with octreotide was investigated, in cancer patients receiving high-dose cisplatinum. The effects of two different regimens of octreotide were employed: (a) 250 micrograms sc 1 h before and 1 h after cisplatinum, or (b) at 250 micrograms sc every 8 h the day before and 500 micrograms sc 1 h before and 1 h after cisplatinum. These treatments failed to prevent the emetic response to cisplatinum. In addition, similar increase in the excretion of urinary 5-HIAA were observed in octreotide-treated patients than in control patients receiving high-dose cisplatinum (Table I).

**Table I** Effects of Octreotide on high-dose cisplatinum-induced increases in 5-HIAA excretion

| | | *Time after cisplatinum* | | | |
|---|---|---|---|---|---|
| | *n* | *2–4 h* | *4–6 h* | *6–8 h* | *8–24 h* |
| Control | 22 | $118 \pm 24$ | $190 \pm 25$ | $135 \pm 19$ | $39 \pm 23$ |
| Octeotride | 4 | $79 \pm 39$ | $210 \pm 40$ | $183 \pm 45$ | $22 \pm 18$ |

All patients received high-dose cisplatinum ($\geqslant 50 \text{ mg m}^{-2}$) in a 1 h infusion. Results are shown as percentage of increase above baseline levels of 5-HIAA excretion ($\mu$g of 5-HIAA $\text{mg}^{-1}$ of creatinine) at different times after cisplatinum. Baseline 5-HIAA/creatinine excretion averaged: $7.0 \pm 0.6$ for controls and $5.8 \pm 1.3$ for octeotride ($P > 0.1$).

### Discussion

In the present study we evaluated the changes in serotonin and in serotonin metabolism produced by cancer chemotherapeutic drugs with the purpose of understanding the mechanisms by which these agents induce nausea and vomiting. The experiments were conducted in cancer patients scheduled to receive their first course of chemotherapy. Chemotherapy-naive patients were selected to avoid the possible interference of anticipatory emesis, and any effect that repeated cycles of treatment could have on intestinal serotonin and on serotonin metabolism. The results and discussion were based on the primary chemotherapeutic agent; however, most patients received treatment with more than one drug. Consequently, although the changes observed in serotonin metabolism and the vomiting could be due to the primary agent, the contribution of other agents cannot be ruled out. However, in our design, the emetogenic activity of the associated drugs was less than that of the primary agent.

The observation that depletion of serotonin prevents cisplatinum-induced vomiting (Barnes *et al.*, 1987), and that antagonists of serotonin-type 3 receptors are effective against radiation- and chemotherapy-induced emesis (Miner & Sanger, 1986; Costall *et al.*, 1986; Smith *et al.*, 1986; Stables *et al.*, 1987; Fozard, 1987; Andrews *et al.*, 1988; Hawthorn *et al.*, 1988; Cubeddu *et al.*, 1990*a*), suggests that serotonin plays a critical role in the emetic response to these treatments. The present study provides further evident of the role of serotonin in chemotherapy-induced nausea and vomiting. First, two strongly emetogenic cytotoxic drugs, high-dose cisplatinum and dacarbazine, induced marked increases in the metabolism of serotonin in a time course that paralleled the onset of nausea and vomiting. Both high-dose cisplatinum and dacarbazine induce an early (2–3 h after chemotherapy) and intense emetic response of 4 to 6 h duration (Martin-Jimenez y Diaz-Rubio, 1985; Gralla *et al.*, 1981; Cubeddu *et al.*, 1990*a*; present study). For high-dose cisplatinum and for dacarbazine, the comparable time courses for emesis and for the increase in serotonin metabolism suggests a cause-effect relationship between serotonin and the emetic response. Second, two chemotherapeutic drugs with mild to moderate emetic activity, cyclophosphamide and low-dose cisplatinum, produced smaller and less consistent increases in serotonin metabolism than the strongly emetogenic cytotoxics; therefore, a temporal relationship between the emetic response and the urinary excretion of 5-HIAA could not be determined for these two treatments. Contrary to cisplatinum and dacarbazine, cyclophosphamide does not produce early emesis (Fetting *et al.*, 1982; Cubeddu *et al.*, 1990*b*), nor does it produce an early increase in 5-HIAA (present study). The results of this study suggest that the amount of serotonin released and the time course of the release determines the severity, the time of onset and the pattern of emesis induced by a specific chemotherapeutic drug. If a cytotoxic drug induces a large release of serotonin within a short period of time, an intense period of vomiting would be expected for this drug, associated to large increases in urinary excretion rate of 5-HIAA. However, if lower amounts of serotonin are released or even if the total amount of serotonin released is similar to that produced by high-dose cisplatinum, but the release occurs over a long period of time (i.e., 24 h), only mild to moderate vomiting spread out over the time of serotonin release would develop. In addition, significant increases above baseline in urinary 5-HIAA excretion may not be observed since the amount of serotonin released will be diluted over many hours.

Information about the source of serotonin released by chemotherapeutic drugs can be obtained from data on the normal distribution of serotonin in the body. In humans, nearly 80% of the serotonin is in the gastrointestinal tract (6–9 mg), 95% of which is within enterochromaffin, as well as in other enteroendocrine cells. The rest of the body serotonin is divided between platelets (1–2 mg), and other tissues, including the CNS (1 mg) (Feldberg & Toh, 1953; Erspamer, 1954; Resnick & Gray, 1961). High-dose cisplatinum and dacarbazine releases approximately 1 to 3 mg of serotonin over a period of 6 h. Thus, if serotonin were to be released from platelets, a complete emptying of all serotonin platelet content would be required in order to account for the changes in 5-HIAA observed after high-dose cisplatinum or dacarbazine. However, in the present study we demonstrated that neither high-dose cisplatinum, nor cyclophosphamide affect the platelet serotonin content. Consequently, the most likely source of the increase in 5-HIAA is the enterochromaffin and enteroendocrine cells of the gastrointestinal tract. These findings support previous observations indicating that urinary 5-HIAA is a marker of gastrointestinal serotonin content and turnover (Erspamer & Testini, 1959; Bertaccini, 1960). Therefore, an increase in urinary 5-HIAA excretion (in the absence of carcinoid tumoral cells) should reflect increases in turnover (increase synthesis and/or release) of gastrointestinal serotonin. Interestingly, in ferrets, cisplatinum increases the gastrointestinal turnover of serotonin (Gunning *et al.*, 1987). In summary, these findings suggest that high-dose cisplatinum and dacarbazine, and probably cyclophosphamide, release serotonin from the enterochromaffin cells of the gastrointestinal tract.

Once released, serotonin could act locally or pass into the circulation in order to induce nausea and vomiting. The contribution of plasma serotonin was explored in this investigation. However, neither platelet serotonin nor free plasma serotonin increased significantly during the period of intense emesis after high-dose cisplatinum, suggesting that most of the serotonin released from enterochromaffin cells is converted to 5-HIAA within the gut wall and/or is released from the gut into the portal system and converted to 5-HIAA on its passage through the liver (a first pass extraction has been demonstrated for serotonin). These results suggest that it is the free serotonin within the gut wall and not the circulating serotonin that plays an active role in emesis induced by chemotherapeutic drugs. This is consistent with the clinical observation that patients with carcinoid tumours may have very high concentrations serotonin in plasma, yet do not experience the intense emesis observed in cisplatinum or dacarbazine-treated patients (Martin-Jimenez & Diaz-Rubio, 1985; Kvols, 1988). In favour of a local, intestinal site of action for serotonin, is the observation that visceral denervation of the ferret, prevents cisplatinum-induced vomiting in this animal (Hawthorn *et al.*, 1988).

Presently, the mechanism by which chemotherapeutic agents induce the release of serotonin is unknown. The presence of receptors on enterochromaffin and in enteroendocrine cells for each of the cytotoxics is unlikely, rather a mechanism related to their cytotoxicity is more plausible. The drugs with strongest emetic activity act as aklylating agents, inhibit DNA biosynthesis and kill cells in all stages of the cell cycle. Rapid cell killing may release substances from dying cells that induce serotonin release from enteric serotonin-containing cells, the cytotoxics may have a specific direct damaging effect on the serotonin cells and/or produce severe mucosal damage and an unspecific damage of the serotonin-containing cells leading to serotonin release. If the release occurs through a normal secretory process, it would be sensitive to somatostatin, a well known inhibitor of hormonal secretion (Katz & Erstad, 1989; Reichlin, 1985) and of serotonin release from carcinoid tumours, which are neoplasms of enterochromaffin cells (Kvols, 1988). Octreotide, a stable long-acting analog of the peptide hormone somatostatin, reduces clinical symptoms, inhibits serotonin release and decreases urinary levels of 5-HIAA in carcinoid patients (see Kvols, 1988 for review). However, with the two treatment schedules employed the somatostatin analog failed to prevent both the emetic response as well as the increases in 5-HIAA induced by high-dose cisplatinum in cancer patients (present study). These results suggest that cisplatinum in high doses induces serotonin release by a mechanism different of the normal secretory hormonal release, and of that observed in tumoral enterochromaffin cells. Studies in experimental animals revealed a severe mucosal damage of the ileum and jejunum after high-dose cisplatinum, and that the extent of the damage was related to the severity of emesis (Gunning *et al.*, 1987). Consequently, although the precise mechanism of cytotoxic-induced gastrointestinal serotonin release is unclear, it seems to be through a direct or indirect lesion of the enterochromaffin cells.

In summary, the serotonin hypothesis could be stated as follows: 'chemotherapeutic drugs and radiotherapy induces the release of serotonin from enterochromaffin and/or other enteroendocrine cells perhaps by damage of the gut mucosa, the released serotonin would activate 5-HT3 receptors on visceral afferent fibres increasing afferent input to the brain, stimulating the chemoreceptor trigger zone and/or vomiting centre with the consequent production of nausea and emesis. The serotonin released would be metabolised within the intestine and/or on its passage through the liver, leading to increases both in plasma and urine of its main metabolite, 5-HIAA. The changes in 5-HIAA would reflect the amount of serotonin released and the severity and pattern of the emetic response'. From a teleological point of view, chemotherapeutic drugs or radiotherapy-produced emesis, may represent an old-learned, life-saving reflex to eliminate ingested toxins that damage the gastrointestinal mucosa. The reflex is triggered even if drugs or agents that damage the gut mucosa are administered by routs other than the oral, such as in the case of chemotherapeutic agents and irradiation therapy.

Although we have oriented our discussion in favour of a peripheral mechanism for serotonin-induced emesis, there is strong evidence in favour of a central site of action for serotonin and serotonin antagonists. For example, central administration of cisplatinum has been reported to induce vomiting in dogs (Smith *et al.*, 1988) and the central administration of 5-HT3 antagonists prevents vomiting induced by cisplatinum (Higgins *et al.*, 1989). The largest concentration of 5-HT3 receptors is present in the brain medulla in the nucleus tractus solitarious and area postrema regions, where the chemoreceptor trigger zone is located and where the vagal afferents enter the brain (Kilpatrick *et al.*, 1989). On this line, we may add that patients receiving low doses of cisplatinum or cyclphosphamide-based treatments showed either small or no increases in urinary 5-HIAA excretion, nevertheless they experienced mild to moderate emesis, and their emesis was controlled by 5-HT3 antagonists (Cubeddu *et al.*, 1990*b*). One possibility is that measurements of urinary 5-HIAA may not be sensitive enough to detect small increases in serotonin release induced by cytotoxics with mild to moderate emetic activity. However, it is also possible that the nausea and vomiting associated to low-dose cisplatinum and cyclophosphamide-based treatments, is mediated mainly through central mechanisms; whereas that induced by high-dose cisplatinum and dacarbazine may be triggered by peripheral as well as by central mechanisms. Thus, serotonin and 5-HT3 receptors play a role both at the periphery as well as in the CNS, and the relative contribution of peripheral and central sites may vary with the type of cytotoxic agent employed, its dose and mechanism of action.

The authors are indebted to Indhira Garcia, Victdoris Gonzalez and Maria Andrade for their valuable assistance in the conduction of the study. The authors would also like to thank colleagues, personnel and facilities of the Hospital Luis Razetti, Hospital Padre Machado and Hospital Domingo Luciani where the patients were treated and the clinical studies conducted.

This work was supported by a Grant from the Consejo de Desarrollo Cientifico y Humanistico of the UCV, No. 07-10-2441-90 to Dr Luigi X. Cubeddu.

### References

ANDREWS, R.L.R., RAPEPORT, W.G. & SANGER, G.J. (1988). Neuropharmacology of emesis induced by anti-cancer therapy. *Trends in Pharmacol. Sci.*, **9**, 334–341.

BARNES, N.M., BARRY, J.M., COSTAL, B., NAYLOR, R.J. & TATTERSALL, F.D. (1987). Antagonism by parachlorophenylalanine of cisplatin-induced emesis. *Br. J. Pharmacol.*, **92**, 649P.

BERTACCINI, G. (1960). Tissue 5-hydroxytryptamine and urinary 5-hydroxyindoleacetic acid after partial or total removal of the gastro-intestial tract in the rat. *J. Physiol.*, **153**, 239–249.

COSTALL, B., DOMENEY, A.M., NAYLOR, R.J. & TATTERSALL, F.D. (1986). 5-hydroxytryptamine M-receptor antagonism to prevent cisplatinum-induced emesis. *Neuropharmacology*, **25**, 959–961.

CUBEDDU, L.X., HOFFMANN, I.S., FUENMAYOR, N.T. & FINN, A.L. (1990*a*). Efficacy of ondansetron (GR 38032F) and the role of serotonin in cisplatin induced nausea and emesis. *N. Engl. J. Med.*, **322**, 810–816.

CUBEDDU, L.X., HOFFMANN, I.S., FUENMAYOR, N.T. & FINN, A.L. (1990*b*). Antagonism of serotonin S3 receptors with ondansetron prevents nausea and emesis induced by cyclophosphamide-containing chemotherapy regimens. *J. Clin. Oncol.*, **8**, 1721–1727. (1990*a*), **322**, 810–816.

ENCK, R.E. (1977). Mallory-Weiss lesion following cancer chemotherapy. *Lancet*, **1**, 927.

ERSPAMER, V. (1954). Il sistema cellulare enterocromaffine e l'enteramina (5-idrossitriptamina). *R. C. Sci. Farmitalia*, **1**, 1–193.

ERSPARMER, V. & TESTINI, A. (1959). Observations on the release and turnover rat of 5-hydroxytryptamine in the gastrointestinal tract. *J. Pharm. Pharmacol.*, **11**, 618–623.

FELDBERG, W. & TOH, C.C. (1953). Distribution of 5-hydroxytryptamine (serotonin, enteramine) in the wall of the digestive tract. *J. Physiol.*, **119**, 352–362.

FETTING, J.H., GROCHOW, L.B., FOLSTEIN, M.S., ETTINGER, D.S. & COLVIN, M. (1982). The course of nausea and vomiting after high-dose cyclophosphamide. *Cancer Treat. Rep.*, **66**, 1487–1493.

FOZARD, J.R. (1987). 5-HT3 receptors and cytotoxic drug-induced vomiting. *Trends in Pharmacol. Sci.*, **8**, 44–45.

GRALLA, R.J., ITRI, L.M. & PISKO, S.E. (1981). Antiemetic efficacy of high-dose metoclopramide: randomized trials with placebo and prochlorperazine in patients with chemotherapy-induced nausea and emesis. *New Engl. J. Med.*, **305**, 905–909.

GRALLA, R.J., TYSON, L.B., KRIS, M.G. & CLARK, R.A. (1987). The management of chemotherapy-induced nausea and vomiting. *Med. Clin. North Amer.*, **71**, 289–301.

GUNNING, S.J., HAGAN, R.M. & TYERS M.B. (1987). Cisplatin induced biochemical and histological changes in the small intestine of the ferret. *Br. J. Pharmacol.*, **90**, 135P.

HAWTHORN, J., OSTLER, K.J. & ANDREWS, P.L.R. (1988). The role of the abdominal visceral innervation and 5-hydroxytriptamine M-receptors in vomiting induced by the cytotoxic drugs cyclophosphamide and cisplatin in the ferret. *Quart. J. Exper. Physiol.*, **73**, 7–21.

HIGGINS, G.A., KILPATRICK, G.J., BUNCE, K.T., JONES, B.J. & TYERS, M.B. (1989). 5-HT3 receptor antagonists injected into the area postrema inhibit cisplatin-induced emesis in the ferret. *Br. J. Pharmacol.*, **97**, 247–255.

KATZ, M.D. & ERSTAD, B.L. (1989). Octreotide, a new somatostatin analogue. *Clin. Pharm.*, **8**, 255–273.

KILPATRICK, G.H., JONES, B.J. & TYERS, M.B. (1989). Binding of the 5-HT3 ligand, 3H-GR65630, to tar area postrema, vagus nerve and the brains of several species. *Eur. J. Pharmacol.*, **159**, 157–160.

KVOLS, L.K. (1988). The carcinoid syndrome: a treatable malignant disease. *Oncology*, **2**, 33–39.

LASZLO, J. & LUCAS, V. (1981). Emesis as a critical problem in chemotherapy. *N. Engl. J. Med.*, **305**, 948–994.

LAZLO, J. (1983). Nausea and vomiting as major complications of cancer chemotherapy. In *Drugs*, Adis Press Ltd: Auckland, N.Z. **25**, (Suppl. 1): 1–7.

LEIBUNDGUT, U. & LANCRANJAN, I. (1987). First results with ICS 205-930 (5-HT, receptor antagonist). *Lancet*, **(May)**, 1198.

MARTIN-JIMENEZ, M. & DIAZ-RUBIO, E. (1985). Curso espontaneo de la emesis inducida por cisplatino y evaluacion preliminar de la metoclopramida en dosis bajas intravenosas. *Oncologia*, **IX**, 223–228.

MARTIN-JIMENEZ, M., DIAZ-RUBIO, E., SANGRO, B. & MAROTE, R. (1988). Laparotomic eventration of colonic prolapse after chemotherapy-induced emesis. *J. Surg. Oncol.*, **37**, 204.

MARTY, M., POUILLART, P., SCHOLL, S. & others (1990). Comparison of the 5-hydroxytryptamine-3 (serotonin) antagonist ondansetron (GR 38032F) with high-dose metoclopramide in the control of cisplatin-induced emesis. *N. Engl. J. Med.*, **322**, 816–821.

MENDELSOHN, J. (1987). Principles of neoplasia. In *Principle of Internal Medicine*, Braudwald, E., Isselbacher, K.J., Petersdorf, R.G., Wilson, J.D., Martin, J.B. & Fauci, A.S. McGraw Hill, 11th edition, p. 429.

MINER, W.D. & SANGER, G.J. (1986). Inhibition of cisplatin-induced vomiting by selective 5-hydroxytryptamine M-receptor antagonism. *Br. J. Pharmacol.*, **88**, 497–499.

RESNICK, R.H. & GRAY, S.J. (1961). Distribution of serotonin (5-hydroxytryptamine) in the human gastrointestinal tract. *Gastroenterology*, **41**, 119–121.

SMITH, W.L., JACKSON, C.B., PROAKIS, A.G., LEONARD, C.A., MUNSON, H.R. & ALPHIN, R.S. (1986). Zacopride: a unique and potent inhibitor of cancer-chemotherapy-induced emesis in dogs. *Proc. Am. Soc. Clin. Oncol.*, **5**, 1017.

SMITH, W.L., CALLAHAM, E.M. & ALPHIN, R.S. (1988). The emetic activity of centrally administered cisplatin in cats and its antagonism by zacopride. *J. Pharm. Pharmacol.*, **40**, 142–145.

STABLES, R., ANDREWS, P.L., COSTALL, B. & others (1987). Antiemetic properties of the 5HT3 antagonist, GR38032F. *Anticancer Drug Design*, **2**, 97.

RESNICK, R.H. & GRAY, S.J. (1961). Distribution of serotonin (5-hydroxytryptamine) in the human gastrointestinal tract. *Gastroenterology*, **41**, 119–121.

REICHLIN, S. (1985). Somatostatin: parts one and two. *N. Engl. J. Med.*, **309**, 2741-1501, 1556-1563.